# Evaluation and Comparison of the *In Vitro* Cytotoxic Activity of *Withania somnifera* Methanolic and Ethanolic Extracts against MDA-MB-231 and Vero Cell Lines

**DOI:** 10.3797/scipharm.1507-13

**Published:** 2015-09-26

**Authors:** A. N. Srivastava, Rumana Ahmad, Mohsin Ali Khan

**Affiliations:** 1 Dept. of Pathology, Era’s Lucknow Medical College & Hospital, Sarfarazganj, Hardoi Road, Lucknow-226003, India; 2 Chairman Research, Era’s Lucknow Medical College & Hospital, Sarfarazganj, Hardoi Road, Lucknow-226003, India

**Keywords:** Anticancer, Cytotoxicity, MTT assay, *In vitro*, MDA-MB-231, Vero, Breast cancer, Withaferin A, *Withania somnifera*

## Abstract

*Withania somnifera* Dunal (WS), commonly known as Ashwagandha in India, belongs to the family Solanaceae. It is extensively used in most of the Indian herbal pharmaceuticals and nutraceuticals. In the current study, the *in vitro* cytotoxic activity of methanolic, ethanolic, and aqueous extracts of WS stems was evaluated using cytometry and the MTT assay against the MDA-MB-231 human breast cancer cell line. Methanolic and ethanolic extracts of WS showed potent anticancer activity on the MDA-MB-231 human breast cancer cell line, whereas the aqueous extract did not exhibit any significant activity at 100 µg/ml. The percentage viability of the cell lines was determined by using the Trypan blue dye exclusion method. Cell viability was reduced to 21% and 0% at 50 and 100 µg/ml of the methanolic extract, respectively, as compared to 19% and 0% at 50 and 100 µg/ml for the ethanolic extract and 37% at 100 µg/ml in sterile Milli-Q water after 48 hours of treatment. Methanolic and ethanolic extracts of WS were shown to possess IC_50_ values of 30 and 37 µg/ml, respectively, by the MTT assay and cytometer-based analysis, with the methanolic extract being more active than the other two. On the other hand, methanolic and ethanolic extracts of WS did not exhibit any significant *in vitro* activity against the normal epithelial cell line Vero at 50 µg/ml. HPLC was carried out for the analysis of its phytochemical profile and demonstrated the presence of the active component Withaferin A in both extracts. The methanolic and ethanolic extracts of *Withania* should be studied further for the isolation and characterization of the active components to lead optimization studies.

## Introduction

Breast cancer is one of the leading causes of death in women [1]. Recent reports indicate that breast cancer is emerging as a prevalent cancer amongst women, surpassing cervical cancer in India [2]. Several chemotherapeutic drugs have been identified to treat breast cancer, yet a convincing cure remains elusive. Therefore, there is a continuing need for development of new anticancer drugs. Natural products have played an important role in drug discovery and have formed the basis of most early medicines. Drug discovery from natural products has led to the isolation of highly active anticancer agents [3]. Isolation and characterization of pharmacologically active compounds from medicinal plants continue to date. India is one of the richest biodiversity centers with respect to medicinal plants. Such plants are utilized in the traditional system of medicine (Ayurveda) for cancer treatment. Plant-derived compounds have played an important role in the development of several clinically useful anticancer agents [3].

*Withania somnifera* (Ashwagandha) is an annual herb growing in dry and arid soil as a wild plant [4] and is well-described in Ayurveda, the ancient Indian system of plant medicine for immunomodulation and anti-aging [5]. *In vitro* anticancer activity of the root, stem and leaves of WS has been evaluated [6]. Thus, WS has been found to have anti-inflammatory [7], antitumour and radiosensitizing actions [8, 9], and analgesic activity [10].

Research on animal cell cultures has shown that the herb decreases the levels of the nuclear factor kappa B, suppresses the intercellular tumor necrosis factor, potentiates apoptotic signaling in cancerous cell lines [11], and also possesses immunomodulatory effects [12]. One of the most exciting of the possible uses of Ashwagandha is its capacity to fight cancers by reducing tumor size [13]. To investigate its use in treating various forms of cancer, the antitumor effects of WS have been evaluated in urethrane-induced lung tumors in adult male mice as well as in skin carcinoma [14]. Following administration of Ashwagandha over a period of seven months, the histological appearance of the lungs of animals which received the herb was found to be similar to those observed in the lungs of control animals. The studies so far indicate that WS could prove to be a good natural source of a potent and relatively safe radiosensitizer/chemotherapeutic agent in lung cancer cases as it causes an increase in serum immunoglobulin levels and increased CD3^+^ and CD8^+^ expression. The current study was an attempt to investigate the *in vitro* anticancer potential of WS on the human breast cancer cell line. The selection of the plant was based on valuable information obtained from Ayurveda on anticancer properties and detailed ethno-botanical reviews. However, detailed investigations on the anticancer properties of WS have not been done. For this purpose, we chose to study the anticancer activity of WS on human breast cancer cells in detail. The data suggest that WS methanolic and ethanolic extracts exhibited potent cytotoxic activity with IC_50_ values less than 100 μg/ml.

## Results and Discussion

### Methanolic and Ethanolic Extracts of WS Showed Cytotoxicity against Human Breast Cancer Cells with Less of an Effect on Normal Cells

**Fig. 1:**
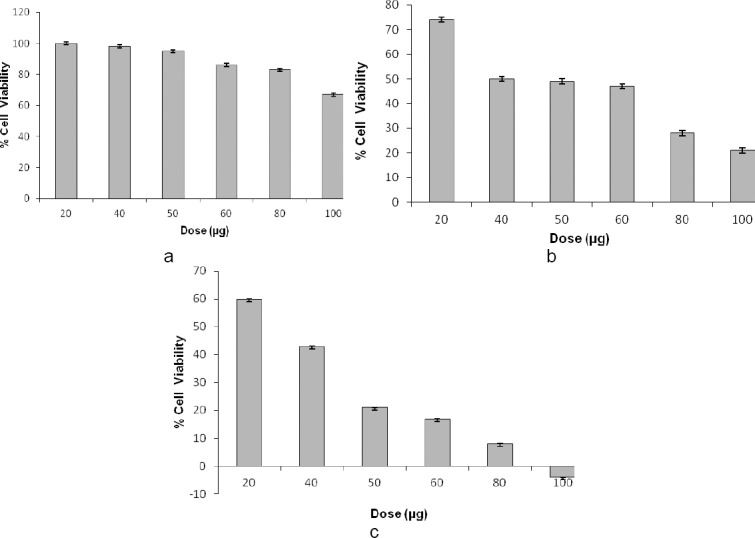
Dose-dependent effect of WS methanolic extract in 1% (a), 10% (b), and 50% (c) DMSO on the viability of MDA cells *in vitro*. The final concentration of DMSO in each well did not exceed 0.5% (v/v).

**Fig. 2:**
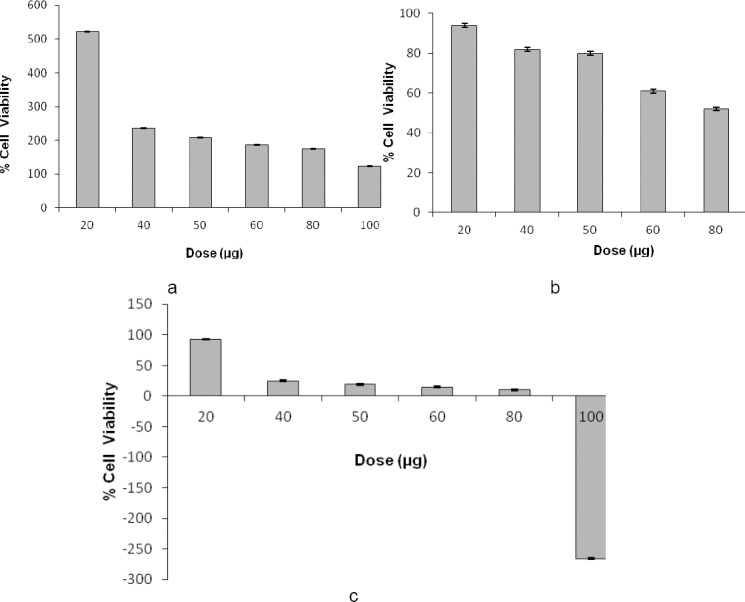
Dose-dependent effects of WS ethanolic extract in 1% (a), 10% (b), and 50% (c) DMSO on the viability of MDA cells *in vitro*. The final concentration of DMSO in each well did not exceed 0.5% (v/v).

Different solvents were tried for better dissolution and cytotoxic effects of methanolic and aqueous extracts of *Withania* against the MDA cell line viz. 10% DMSO, 50% DMSO, and sterile water. It was found that though the methanolic and aqueous extracts of *Withania* were both soluble in water, the cytotoxic effects of methanolic and aqueous extracts of *Withania* in sterile water were only 37% and 17%, respectively, at 100 µg/ml. Table 1 shows the cytotoxicity of various extracts of WS. Results indicated that both methanolic and ethanolic extracts of WS possessed dose-dependent cytotoxic effects against human breast cancer cells. Figures 1 and 2 depict the dose-dependent effects of methanolic and ethanolic extracts in 1%, 10%, and 50% DMSO on MDA cells using the MTT assay.

**Tab. 1:**
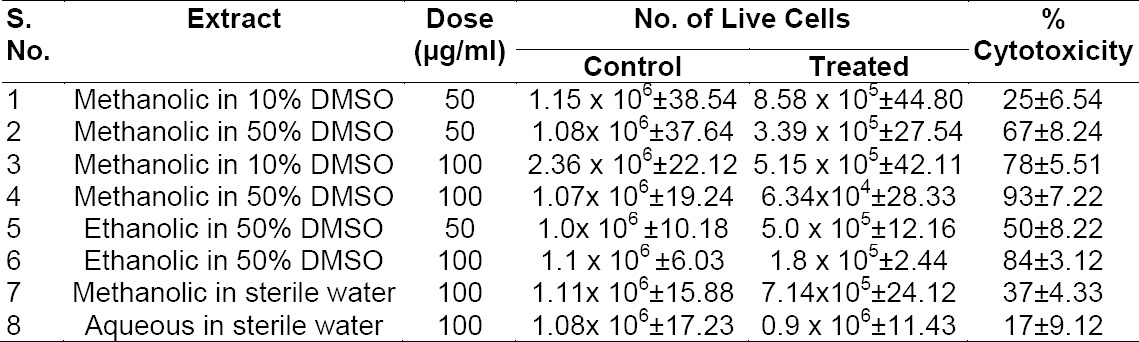
Evaluation of Cytotoxicity of WS Extract

**Fig. 3:**
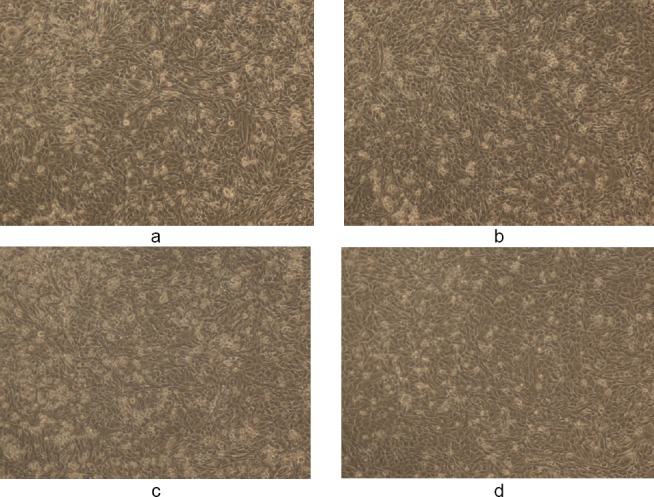
(a, c) Controls showing untreated MDA human breast cancer cells in 1% DMSO and (b, d) the cytotoxic activity of WS methanolic extract at 50 and 100 µg/ml, respectively, in 1% DMSO after 48 h (magnification 10X). The final concentration of DMSO in each well did not exceed 0.5% (v/v).

**Fig. 4:**
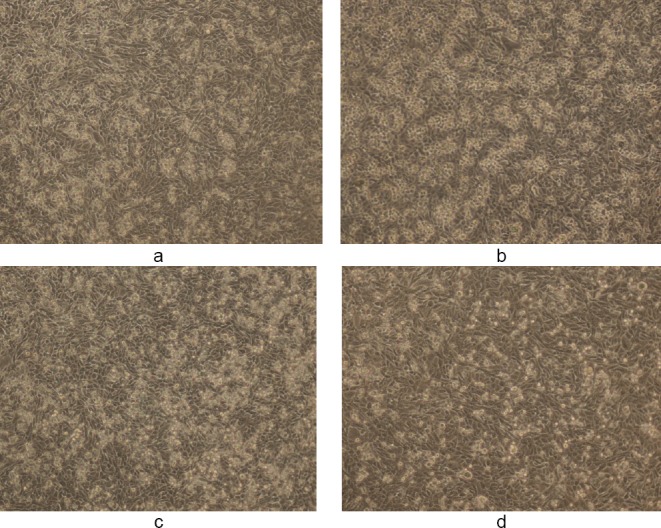
(a, c) Controls showing untreated MDA human breast cancer cells in 10% DMSO and (b, d) the cytotoxic activity of WS methanolic extract at 50 and 100 µg/ml, respectively, in 10% DMSO after 48 h (magnification 10X). The final concentration of DMSO in each well did not exceed 0.5% (v/v).

**Fig. 5:**
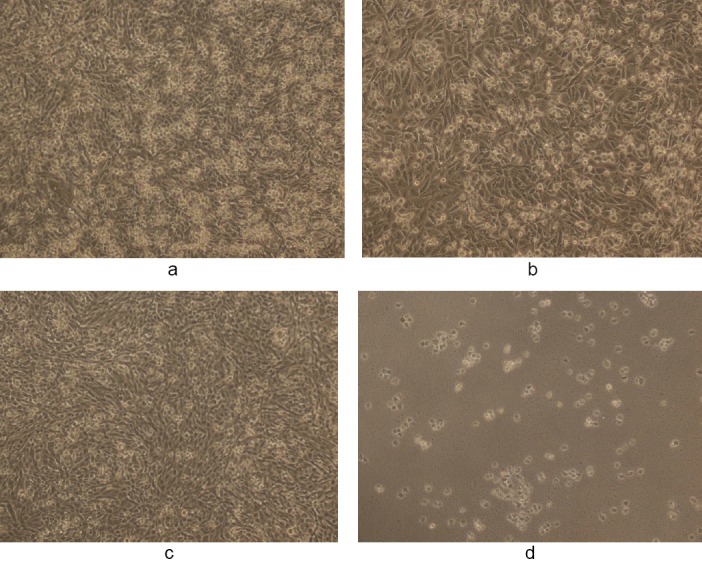
(a, c) Controls showing untreated MDA human breast cancer cells in 50% DMSO and (b, d) the cytotoxic activity of WS methanolic extract at 50 and 100 µg/ml, respectively, in 50% DMSO after 48 h (magnification 10X). The final concentration of DMSO in each well did not exceed 0.5% (v/v).

**Fig. 6:**
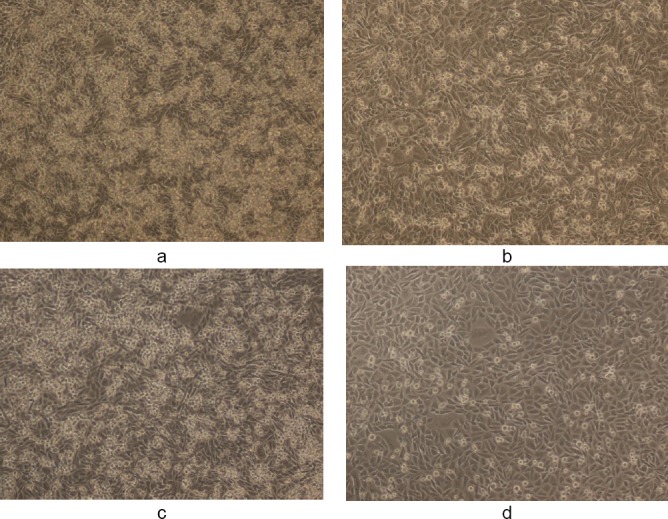
(a, c) Controls showing untreated MDA human breast cancer cells in 1% DMSO and (b, d) the cytotoxic activity of WS ethanolic extract at 50 and 100 µg/ml, respectively, in 1% DMSO after 48 h (magnification 10X). The final concentration of DMSO in each well did not exceed 0.5% (v/v).

**Fig. 7:**
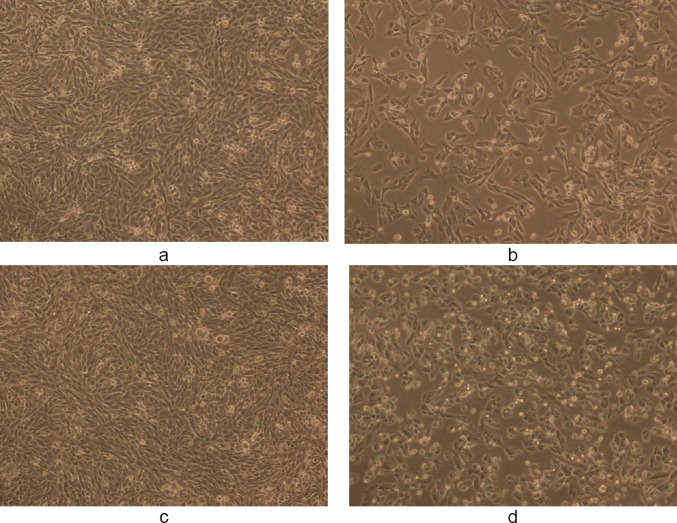
(a, c) Controls showing untreated MDA human breast cancer cells in 10% DMSO and (b, d) the cytotoxic activity of WS ethanolic extract at 50 and 100 µg/ml, respectively, in 10% DMSO after 48 h (magnification 10X). The final concentration of DMSO in each well did not exceed 0.5% (v/v).

**Fig. 8:**
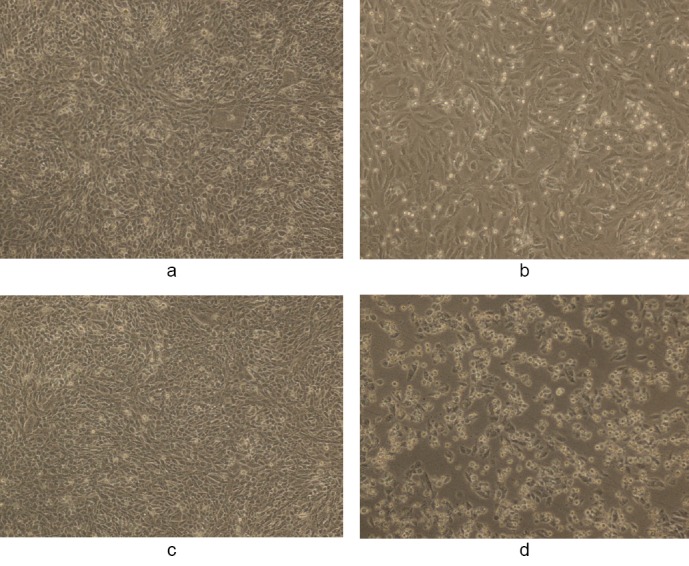
(a, c) Controls showing untreated MDA human breast cancer cells in 50% DMSO and (b, d) the cytotoxic activity of WS ethanolic extract at 50 and 100 µg/ml, respectively, in 50% DMSO after 48 h (magnification 10X). The final concentration of DMSO in each well did not exceed 0.5% (v/v).

Figures 3–8, respectively, depict the morphological analysis of untreated versus treated cells with respect to WS methanolic and ethanolic extracts (50 and 100 µg/ml). It is evident from the figures that both extracts of WS showed cytotoxic effects as well as inhibition of cell proliferation of human breast cancer cells MDA-MB-231, with the methanolic extract being the more effective of the two. Methanolic extract had an IC_50_ of 40 and 30 µg/ml in 10% and 50% DMSO, respectively, as compared to 78 and 37 µg/ml for ethanolic extract in 10% and 50% DMSO, respectively. The treated cells displayed an altered morphology under the inverted microscope. WS caused MDA cells to develop the characteristic features of cell shrinking, rounding, and partial detachment, thus demonstrating the lobulated appearance of apoptotic cells (Figures 5d and 8d).

**Fig. 9:**
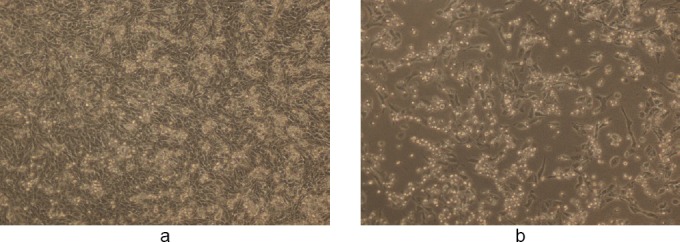
(a) Control showing untreated MDA human breast cancer cells in the presence of 0.5% DMSO and (b) the cytotoxic activity of doxorubicin chloride at 0.5 μM in 0.5% DMSO after 48 h (magnification 10X).

As is evident from the figures, the methanolic and ethanolic extracts did not show any significant activity when 1% DMSO was used as a vehicle. There seemed to be a significant effect of the vehicle on the cytotoxic activity of the extracts, probably due to better dissolution of the extract in 10% and 50% DMSO as compared to 1% DMSO and water. It is also clear from the pictures that DMSO at concentrations of either 0.25% or 0.5% did not have any cytotoxic effect of its own. The positive control, doxorubicin, imparted cytotoxic and dose-dependent inhibition of cell proliferation and the IC_50_ value of doxorubicin on MDA-MB-231 cells was found to be 0.50 ± 0.03 μM (Figure 9).

**Fig. 10:**
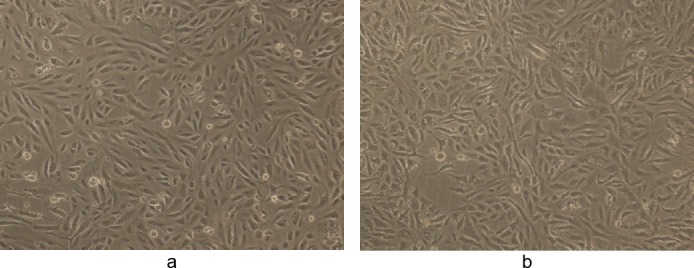
(a) Control showing untreated Vero human kidney epithelial cells in 50% DMSO and (b) the effect of WS methanolic extract at 50 µg/ml in 50% DMSO after 48 h (magnification 10X). The final concentration of DMSO in each well did not exceed 0.5% (v/v).

**Fig. 11:**
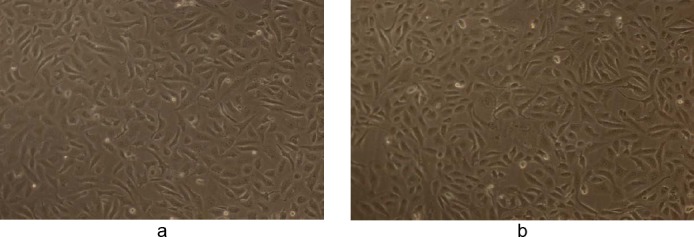
(a) Control showing untreated Vero human kidney epithelial cells in 50% DMSO and (b) the effect of WS ethanolic extract at 50 µg/ml in 50% DMSO after 48 h (magnification 10X). Final concentration of DMSO in each well did not exceed 0.5% (v/v).

The effect of methanolic and ethanolic extracts of WS was also tested on the human normal epithelial cell line Vero at 50 µg/ml. Both extracts did not show any significant cytotoxic effect on the normal cell line (IC_50_ value of >100 μg/ml) at the concentration that was cytotoxic to human breast cancer cells (Figures 10 and 11).

Qualitative phytochemical characterization of extract constituents was carried out using HPLC. From the analysis, the concentration of major constituents, especially Withaferin A, in methanolic extract was found to be higher as compared to ethanolic extract, thus accounting for the higher anticancer activity of the methanolic extract as compared to the ethanolic one (Figures 12b and c). Figure 12 (d) depicts the chromatogram of methanolic and ethanolic extracts co-injected together. Most of the peaks obtained in individual chromatograms of methanolic and ethanolic extracts were retained in the co-injected sample. The analytical HPLC method used in the study provided a good baseline resolution of peaks of withanolides present in WS extracts with reference to the Withaferin A standard (R_t_ = 24.5 min).

**Fig. 12a:**
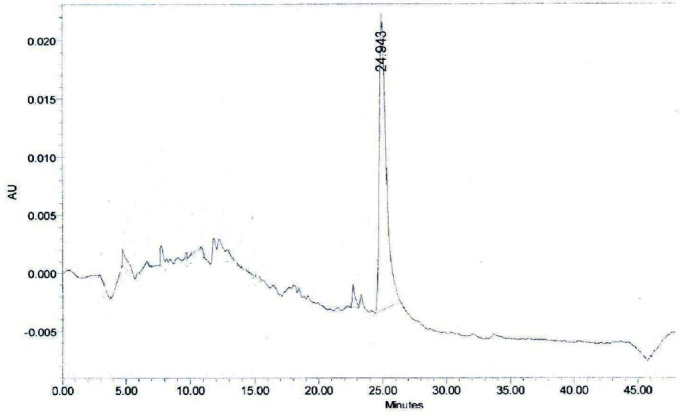
HPLC profile of Withaferin A working standard. Withaferin A, (R_t_ = 24.943 min).

**Fig. 12b:**
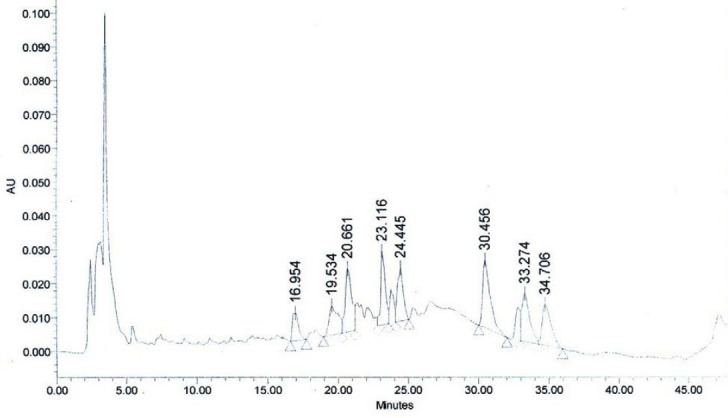
HPLC profile of the methanolic extract of WS stems under identical conditions. Withaferin A, (R_t_ = 24.445 min).

**Fig. 12c:**
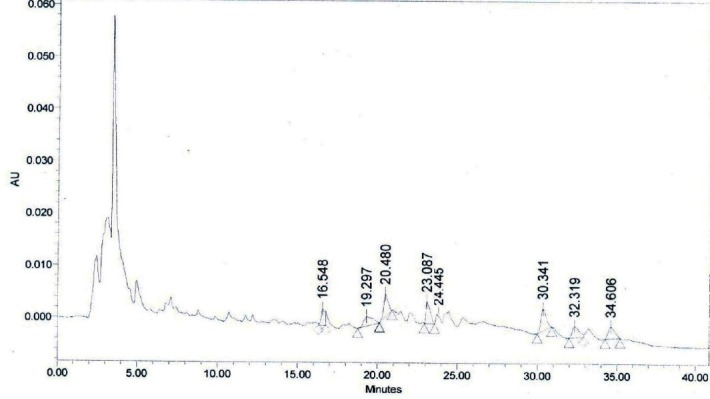
HPLC profile of the ethanolic extract of WS stems. Withaferin A, (R_t_ = 24.445 min).

**Fig. 12d:**
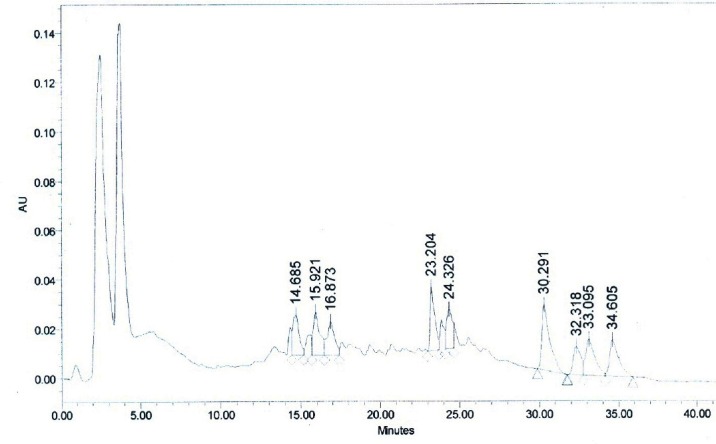
HPLC chromatogram of the co-injection of methanolic and ethanolic extracts of WS stems. Withaferin A, (R_t_ = 24.326 min).

A few studies have reported the anticancer activity of the ethanolic extract of WS against some cell lines, but the cytotoxic activity of the methanolic extract is being reported for the first time. The anticancer activity has been previously reported of WS against various human cell lines, but according to the literature, it is probably being reported for the first time against the human breast cancer cell line MDA. Although the stem of WS is well-known for its importance in Ayurveda, reports on the medicinal activities of the stem are very few [6].

The mechanism of action of the alcoholic extract of WS on cancer cell lines has been reported previously [15]. The ethanolic extract of WS has been shown to induce apoptosis in breast cancer cells. Treatment with WS ethanolic extract has been shown to cause a significant increase in the G2/M phase (indicating cell cycle arrest and blockade of mitosis). Thus, treatment with WS alcoholic extracts induces apoptosis in breast cancer cell lines, as well as inhibition of the cell cycle. DNA fragmentation is a hallmark property of apoptosis [15] and a DNA fragmentation assay has corroborated the fact that the ethanolic extracts of WS induce apoptosis in breast cancer cells [16].

Ashwagandha (WS) is rich in phytochemicals including withanamides, withanolides, withanosides, withanolide glycosides, steroidal saponins, and lignanamides [17–19]. The major constituents of alcoholic extracts of Ashwagandha are steroidal alkaloids and lactones, known as withanolides [20]. Amongst these, Withaferin A and withanone have been demonstrated to be cytotoxic to human cancer cells [21–26]. It has been demonstrated that the alcoholic extract of Ashwagandha leaves and its component withanone cause selective growth arrest of cancer cells through activation of the p53 and ROS signaling pathways [24, 26–28].

Thus, this study revealed the enormous medicinal potential of the stem of this plant. It would be our endeavor in the future to determine the *in vitro* effect of WS alcoholic extracts on a panel of other cancer and normal cell lines as well as *in vivo* studies. This would be a highly desirable trait for a potential therapeutic anticancer agent.

## Materials and Methods

### Reagents

PBS (pH = 7.2, 1X), 0.4% Trypan blue, 0.25% Trypsin-EDTA (1X), DMEM/F-12 (1X) (Dulbecco’s Minimum Essential Medium), and antibiotic (100X) were obtained from Gibco, Life Technologies; whereas FBS and MTT were from Himedia. Doxorubicin hydrochloride solution was purchased from Sigma Chemical Co. (St. Louis, MO, USA). Dimethyl sulfoxide (DMSO) was purchased from Calbiochem. Standard Withaferin A was purchased from Natural Remedies Ltd. Veerasandra, Bangalore-100. All reagents used in the HPLC analysis were of HPLC grade. All other chemicals used in the study were of analytical grade.

### Collection of Plant Material

Fresh stems of WS were collected and dried under shade and then blended into fine powder with a grinder.

### Sample Preparation

Three separate extracts of WS were prepared by extracting 25 g of WS powder with three different solvents viz. water, 50% methanol (1:8), and 50% ethanol (1:8). In each extraction, the upper layer of solvent was collected in a beaker after 24 h and the procedure was repeated thrice in the interval of 24 h continuously till the color of solvent disappeared. All extracts from a single source were pooled together and filtered using Whatman No.1 filter paper (125 mm). The filtered extracts were concentrated at 100°C in a water bath. The semi-solid paste formed was transferred to a Petri plate and kept in a hot air oven till it attained a powdered form. The total weight of powder was measured and stored in an air-tight container for further use. For biological studies, 20 mg of methanolic extract was dissolved in 1%, 10%, and 50% DMSO (Calbiochem) and sterile Milli-Q water, respectively, at a concentration of 20 mg/ml. The extracts were passed through 0.22-µm sterile Millipore syringe filter units (Fisher Scientific) prior to being used in the cell culture studies.

## Biological Evaluation

### Cell Lines

MDA-MB-231 (human breast carcinoma, ER^-^, tumorigenic, and invasive) and Vero (ATCC-CCL-81) normal kidney epithelial cell lines were obtained from the National Centre for Cell Science (NCCS), Pune, India, and as such, were maintained by sub-culturing and passaging as monolayers in 25 and 75 cm^2^ cell culture flasks (Nest, Tarsons) at 37°C in the Tissue and Cell Culture Lab, Era’s Medical College, Lucknow, in an incubator gassed with an atmosphere of 5% CO_2_ at 95% humidity, in advanced Dulbecco’s Minimum Essential Medium (DMEM) containing phenol red as a pH indicator and supplemented with 5% FBS. The medium, prior to being used in cell culture experiments, was vacuum-filtered using a Corning filtration system. The medium required an atmosphere of 5% CO_2_ to produce HCO_3_ buffering capacity to maintain the pH at 7.4 for normal cell growth.

### Cell Culture

For the experiments, cells were trypsinized and cultured in 6-flat-bottomed well plates (Linbro, MP Biomedicals) at a density of 0.5 × 10^5^ cells/well initially for 24 h, so as to allow the cells to attach. After 24 h of incubation, the cells were exposed to 50 and 100 µg/ml of aqueous, methanolic, and ethanolic extracts of WS (in 0.5% DMSO) for the next 48 h. Suitable untreated controls (containing 0.5% DMSO as a vehicle) were also concomitantly employed. Each dose was tested in at least three replicate wells. Results were interpreted as cell viability *versus* a time period graph.

### Morphological Study

For morphological analysis, cells in 6-well plates were observed under a phase contrast microscope & photographed (Nikon Eclipse Ti, Japan).

### Cytotoxicity Assays

#### Trypan Blue Dye Exclusion Assay

A cell suspension was made at a suitable dilution (1.0 × 10^5^ cells/ml) in PBS. Fifty µl of the cell suspension was taken and mixed with an equal volume of 0.4% Trypan blue. The solution was mixed thoroughly and allowed to stand for 5 min at room temperature. Fifty µl of the solution was transferred to a hemocytometer and viable cells were counted as clear cells and dead cells as blue ones. The number of live cells per ml was calculated using the following formula: % viability = (live cell count/total cell count)*100.

#### (Methyl Tetrazolium-MTT Assay) Determination of Optimal Cell Number for the Assay

In order to determine the optimal cell number required for the assay, serial dilutions of MDA-MB-231 (2,000, 4,000, 6,000, 8,000, 12,000, 14,000, 16,000, and 18,000 cells/100 l) were made in cell culture media and seeded in 96-well microtiter tissue culture plates (Linbro, MP Biomedicals). Cells were cultured in a humidified 5% CO_2_ incubator at 37°C for 24 h. At the end of the incubation period, 20 µl of MTT solution (stock concentration, 5.0 mg/ml in PBS) was added to each well and incubated for 4 hours under the same conditions. Thereafter, medium containing MTT was gently replaced by 200 µl of DMSO to dissolve formazan crystals and the absorbance values were read in an ELISA plate reader (Biorad PW41) at 550 nm with a reference wavelength of 630 nm. A graph was plotted with the number of cells in the X-axis and absorbance at 570/630 nm in the Y-axis. The optimal cell density of the cell line corresponding to absorbance values of 0.9 to 1.0 in the assay was selected for MDA-MB-231 to facilitate the measurement of both stimulation and inhibition of cell proliferation within the linear range.

#### Evaluation of Cytotoxicity and Cell Viability

Briefly, MDA-MB-231 cells were trypsinized and resuspended in the culture medium to get a defined cell number for MDA-MB-231 (16,000/100 µl) in a 96-well microtiter tissue culture plate and cultured in a humidified 5% CO_2_ incubator at 37°C for 24 h. Defined concentrations of the extracts in 1%, 10%, and 50% DMSO were freshly prepared in culture media by serial dilution to get final concentrations of 20, 40, 50, 60, and 100 μg/ml (for WS methanolic and ethanolic extracts). Serial dilution was carried out in cell culture media in such a way that the final concentration of DMSO in the wells did not exceed 0.5% (v/v). Three control wells containing medium alone to serve as blanks were also included. After 24 h of incubation, cells were treated with the above-mentioned concentrations of WS extracts in triplicate for 48 h. Doxorubicin hydrochloride, an anticancer drug, was used as a positive control. An equal volume of DMSO was used as a vehicle control. At the end of the treatment, 20 μl of MTT (stock made in PBS at 5.0 mg/ml) reagent was added to each well and incubated further for 4 h. Thereafter, the culture medium was removed and formazan crystals were dissolved in 200 μl of DMSO. The plates were read in a 96-well microplate reader (Biotek-ELx-800) at a wavelength of 570 nm with a reference wavelength of 630 nm. Percentage cell viability (Y-axis) was calculated from absorbance and plotted against the concentration in μg/ml (X-axis). The % cell survival was calculated as = {(A_T_−A_B_) / (A_C_−A_B_)} ×100 where,

A_T_ = Absorbance of the treatment well

A_B_ = Absorbance of the blank

A_c_ =Absorbance of the control well

% cell inhibition = 100 − Cell Survival

The IC_50_ values of the extracts were obtained from the graph as the concentration which decreased the cell viability by 50%.

### Comparison of the Cytotoxic Activity of Extracts

The question of whether WS extract-mediated suppression of cell viability and growth was selective to cancer cells, and not to normal cells, was addressed by determining the growth inhibitory effects of the above-mentioned methanolic and ethanolic extracts on human normal epithelial cells (Vero cell line). Briefly, Vero cells were seeded (14,000/100 µl) in 96-well microtiter tissue culture well plates initially for 24 h and then treated with WS aqueous, methanolic, and ethanolic extracts (50 μg/ml), and doxorubicin (0.5 μM) for the next 48 h. At the end of the treatment, cells were subjected to the MTT assay. The percentage cell viability (Y-axis) was calculated from the absorbance and plotted against concentration in μg/ml (X-axis).

### HPLC Analysis

#### Preparation of the Standard for HPLC Analysis

Ten mg of Withaferin-A working standard were dissolved in 50 ml of methanol (HPLC grade) which was further diluted by dissolving 1.0 ml of this solution to 50 ml methanol. Calibration curves for standard Withaferin A were found to be linear in the range of 0.2–20 µg/ml.

#### Sample Preparation

One mg each of methanolic/ethanolic extracts were accurately weighed and separately dissolved in 1.0 ml each of 0.5% DMSO (HPLC grade). One ml of this solution was further diluted to 50 ml using HPLC grade methanol. Samples and working standard were filtered through 0.45 μm (Millipore) filters. The injection size for the standard and samples was 10 µl each.

#### Procedure

HPLC was performed on a Waters Alliance 515 HPLC system, equipped with two Waters 515 pumps, a Waters Pump Control Module, degasser, injector, and a Waters 2998 photodiode array detector (Waters, Milford, MA, USA). For all separations, an ODS-2 Hypersil C_18_ reversed-phase column (250 × 4.6 mm, 5 µm particle size, maintained at 25°C) was used. The mobile phase consisted of water (solvent A) and acetonitrile (solvent B) which was applied in the following gradient elution for 55 min: 95% A, 5% B for 0–5 min, 85% A, 15% B for 5 min, 60% A, 40% B for 40 min, 10% A, 90% B for 10 min, 50% A, 50% B for 2 min, 95% A, 5% B for 3 min. The flow rate and sample volume were set at 1.0 ml/min and 10 µl, respectively. All separations were monitored at 243 nm.

### Data Interpretation and Statistical Analysis

Absorbance values that were lower than the control wells indicated a reduction in the rate of cell proliferation. Conversely, a higher absorbance value indicated an increase in cell proliferation. Rarely, an increase in proliferation might be offset by cell death; evidence of cell death was inferred from morphological analysis.

Results were expressed as the mean ± SD of experiments done in triplicate.

## Conclusion

This study showed that the methanolic and ethanolic extracts of stems of WS are highly cytotoxic and inhibit growth of the human breast cancer cell line MDA-MB-231. At the same time, they did not seem to have any significant *in vitro* effect on normal kidney epithelial cells.
